# Comparisons of reference curves for femoral neck geometric parameters between Changsha-Chinese women and women of three ethnic groups in the United States

**DOI:** 10.1007/s11657-025-01571-y

**Published:** 2025-07-08

**Authors:** Lin Li, Yi Shen, Hong Zhang, Ru-Chun Dai, Ling-Qing Yuan, Zhi-Feng Sheng, Xi-Yu Wu

**Affiliations:** 1 Department of Endocrinology and Metabolism, The Affiliated Changsha Hospital of Xiangya School of Medicine, Central South University, Changsha, China; 2 Department of Orthopedics, The Second Xiangya Hospital, Central South University, Changsha, China; 3 Department of Metabolism and Endocrinology, Hunan Provincial Key Laboratory of Metabolic Bone Diseases, National Clinical Research Center for Metabolic Diseases, The Second Xiangya Hospital of Central South University, Changsha, China

**Keywords:** Ethnic difference, Femoral neck fracture, Femoral neck geometric parameter, Reference curve

## Abstract

***Summary*:**

Factors underlying ethnic differences in the incidence of femoral fractures are not fully understood. Reference curves of femoral neck geometric parameters (FNGPS) between Changsha-Chinese women and three United States (US) ethnic groups of women varied by ethnicity, age, and measurement parameters. Further investigations might better explain fracture-rate differences.

**Purpose:**

Osteoporotic fractures are associated with race, and the risk of femoral neck fractures is associated with FNGPs. We compared age-related FNGPs of Changsha-Chinese women with those of three ethnic groups of women in the US.

**Methods:**

Data on 4236 Changsha-Chinese women and non-Hispanic Whites (NHWs), non-Hispanic Blacks (NHBs) and Mexican women (aged 20–91 years) from a laboratory database and research literature, were analyzed to measure their FNGPs: outer diameter (OD), cross-sectional area (CSA), averaged cortical thickness (ACT), endocortical diameter (ED), buckling ratio (BR), section modulus (SM), and cross-sectional moment of inertia (CSMI).

**Results:**

The overall means of the OD, ED, SM, and CSMI of the Changsha-Chinese women were significantly lower than those of the NHWs. Their CSA, ACT, SM, and CSMI were significantly lower than that of the NHBs and the age-related reference curves of their CSA, ACT, SM, and CSMI were significantly lower than those of the NHWs, NHBs, and Mexican women. Their OD and ED reference curves were significantly lower than those of the NHWs and NHBs; their OD, CSA, and ACT reference curves were lower than those of the NHWs; and their CSA, ACT, and SM were significantly lower than those of the NHBs. However, their buckling ratio (BR) reference curve was significantly higher than that of the NHWs, NHBs, and Mexican women in the US.

**Conclusion:**

Ethnic differences in the FNGPs of Changsha-Chinese women and US ethnic groups may explain differences in hip-fracture rates among different ethnic groups.

**Supplementary Information:**

The online version contains supplementary material available at 10.1007/s11657-025-01571-y.

## Introduction

Postmenopausal osteoporosis is a common and frequently occurring disease that endangers women’s health. A fragility fracture of the femoral neck is the most serious complication of osteoporosis, given that it has the highest medical costs and mortality rates among fracture types [1, 2] and its reputation as a leading cause of death among the elderly [3–5]. A direct factor in the occurrence of brittle fractures of the femoral neck is a decrease in bone strength, which depends on bone mineral density (BMD), bone quality, and geometric structure. Changes in the geometric structure of the femoral neck with age is a highly genetic trait and an important factor affecting the bone strength of the femoral neck [6–9], which has a significant impact on brittle fractures of the femoral neck. Recent studies have shown that femoral neck geometric parameters (FNGPs) are an important index reflecting the geometric characteristics of the femoral neck and an independent determinant of the risk for a fragility fracture of the femoral neck [10–12]. A large number of epidemiological surveys have reported racial or regional differences in the incidence of fragility fractures of the femoral neck of the same gender [2, 17,13‒]. For example, the incidence of femoral neck fractures in Canadians is lower than that in Americans and Germans. The incidence of femoral neck fractures among people in Beijing, China, is much lower than that among people in Taiwan and Hong Kong. Furthermore, the incidence of fracture among White women is higher than that among Black women, which is associated with differences in femoral geometry between the two races [17].

In order to further understand ethnic differences in female FNGPs and identify reasons for differences in the incidence of femoral neck fractures, we compared age-related reference curves of FNGPs between Changsha-Chinese women and non-Hispanic Whites (NHWs), non-Hispanic Blacks (NHBs), and Mexican women in the United States (US). These FNGPs included the outer diameter (OD), cross-sectional area (CSA), averaged cortical thickness (ACT), endocortical diameter (ED), buckling ratio (BR), section modulus (SM), and cross-sectional moment of inertia (CSMI).

## Methods

### Subjects

A total of 4236 Changsha-Chinese female subjects aged 20‒91 years, with an average age of 48.5 ± 13.6 years, met the study’s inclusion criteria. Among them, 2395 were premenopausal and 1841 were postmenopausal, with an average menopausal age of 49.0 ± 3.59 years and a median menopausal age of 9.0 years. The data on these subjects were retrieved from a reference database of results of our laboratory’s research [10, 18], and the subjects in this database had not been exposed to various disease factors influencing bone metabolism (e.g., chronic kidney disease, chronic liver diseases, parathyroid or thyroid diseases, diabetes, premature ovarian failure or menopause before 40 years of age, hyperprolactinemia, oophorectomy, rheumatoid arthritis, ankylosing spondylitis, malabsorption syndrome, malignant tumors, blood system diseases, or pathological fractures). Subjects exposed to the effects of various drug factors on bone metabolism (e.g., glucocorticoids, estrogens, thyroid hormones, fluoride, bisphosphonates, calcitonin, thiazide diuretics, barbiturates, antiepileptic drugs, vitamin D, and calcium-containing drugs) were also excluded.

The three ethnic groups in the US were NHWs, NHBs, and Mexican women. Their data were obtained from the third National Health and Nutrition Examination Survey report of the US. Relevant data were obtained from the research literature [19]. The age-related femoral neck bone area (BA) and BMD were used to calculate the reference curves of the age-related FNGPs.

### Measurements of FNGPs

The projected BA, bone mineral content (BMC), BMD, and FNGPs in the hip region, including the femoral neck, were measured using dual-energy X-ray absorptiometry (DXA bone densitometry) (Hologic QDR 4500 A and Delphi A; Hologic, Bedford, MA, US). The precision coefficient of variation (RMSCV) of the femoral neck’s BMD measured by this instrument was 1.17%. The long-term (> 17 years) coefficient of variation (CV) of precision in the daily measurements of the lumbar prosthesis model was less than 0.45%. The femoral neck BA, BMC, and BMD of the three groups of women in the US were also measured using the Hologic series bone mineral densitometer [19]. According to the anatomical position of the femoral neck image and the calculation formula in the literature [9, 11,23,20‒] (Fig. S1), various FNGPs were calculated using the femoral neck BA and/or the BMD. These parameters included the OD, CSA, ACT, ED, BR, SM, and CSMI.

### Statistical analysis

SPSS 17.0 software was used for the statistical analysis and mapping. The mean ± standard deviation of height, weight, body mass index (BMI), femoral neck projection of the BA, BMD, and other FNGPs were calculated by age groups stratified by 10-year intervals. The model with the best goodness-of-fit was selected from various mathematical regression models, and the distribution relationship of the various FNGPs with age: the best fitting curve and 95% confidence interval (95% CI) were analyzed. The comparisons of the FNGPs between Changsha-Chinese women and US women of different ethnicities were analyzed using paired *t*-tests with age-related average-fitting reference curves.

## Results

Table 1 shows the average and standard deviation of the sample size, anthropometric indicators, femoral neck BA, BMC, and BMD of the Changsha-Chinese women in the age groups. The average height of the subjects reached its maximum value at 20–29 years of age, and it gradually decreased with age. The means of the subjects’ body weight, femoral neck BA, and BMC peaked at 40–49 years old, and the mean of the subject’s femoral neck BMD peaked at age 30–39 years and then decreased gradually with increasing age. Significant differences in trends of these indicators were observed among the age groups (all *p* < 0.001).

**Table 1:** Age distributions and anthropometric features of Changsha-Chinese women

Age (yr)	*n*	Weight (kg)	Height (cm)	BMI (kg/m^2^)	FN-BA (cm^2^)	FN-BMC (g)	FN-BMD (g/cm^2^)
20–29	396	50.9 ± 6.35	158.1 ± 5.41	20.4 ± 2.19	4.58 ± 0.33	3.58 ± 0.49	0.783 ± 0.096
30–39	698	54.5 ± 7.78	157.1 ± 5.26	22.0 ± 2.77	4.66 ± 0.31	3.68 ± 0.53	0.790 ± 0.104
40–49	1128	57.7 ± 7.40	156.9 ± 5.13	23.4 ± 2.88	4.86 ± 0.22	3.74 ± 0.52	0.770 ± 0.102
50–59	1102	57.1 ± 7.81	155.2 ± 5.00	23.7 ± 2.99	4.71 ± 0.30	3.29 ± 0.51	0.699 ± 0.100
60–69	638	55.8 ± 8.23	152.7 ± 5.37	23.9 ± 3.14	4.61 ± 0.32	2.87 ± 0.44	0.623 ± 0.091
70–79	228	53.9 ± 9.18	150.6 ± 5.11	23.8 ± 3.80	4.58 ± 0.30	2.66 ± 0.46	0.582 ± 0.105
≥ 80	46	47.6 ± 9.63	148.4 ± 4.98	21.5 ± 3.72	4.61 ± 0.36	2.35 ± 0.46	0.514 ± 0.098

Trends in differences between different age groups in various indicators, all *p* < 0.001

Table 2 shows the mean and standard deviation of the age-related FNGPs in the Changsha-Chinese women. The means of the OD, CSA, ED, SM, and CSMI of the femur neck were highest at 40‒49 years, and the mean of the ACT was highest at age 30–39 years. The ACT changes began at approximately 40 years of age, and the CSA, SM, and CSMI changes, which began at approximately 50 years of age, decreased with age. Only the BR increased with age, and the BR mean was largest in the ≥ 80 years of age group.

**Table 2:** Age-related femoral neck geometric parameters (mean ± SD) in Changsha-Chinese women

Age (yr)	*n*	OD (cm)	CSA (cm^2^)	ED (cm)	ACT (cm)	BR	SM (cm^3^)	CSMI (cm^4^)
20–29	396	3.05 ± 0.22	2.27 ± 0.31	2.75 ± 0.23	0.150 ± 0.019	10.4 ± 1.62	1.23 ± 0.22	1.89 ± 0.46
30–39	698	3.11 ± 0.21	2.34 ± 0.34	2.81 ± 0.21	0.151 ± 0.021	10.5 ± 1.66	1.29 ± 0.23	2.02 ± 0.47
40–49	1128	3.24 ± 0.14	2.37 ± 0.33	2.94 ± 0.15	0.147 ± 0.021	11.3 ± 1.71	1.37 ± 0.20	2.23 ± 0.41
50–59	1102	3.14 ± 0.20	2.09 ± 0.33	2.88 ± 0.20	0.133 ± 0.020	12.1 ± 2.02	1.18 ± 0.22	1.87 ± 0.44
60–69	638	3.07 ± 0.21	1.82 ± 0.28	2.83 ± 0.22	0.118 ± 0.018	13.4 ± 2.52	1.02 ± 0.19	1.57 ± 0.38
70–79	228	3.05 ± 0.20	1.68 ± 0.30	2.83 ± 0.22	0.110 ± 0.021	14.5 ± 3.62	0.94 ± 0.17	1.44 ± 0.32
≥ 80	46	3.07 ± 0.24	1.49 ± 0.29	2.86 ± 0.23	0.097 ± 0.019	16.6 ± 4.32	0.84 ± 0.18	1.30 ± 0.35

Trends in differences between different age groups in various indicators, all *p* < 0.001

**Fig. 1:**
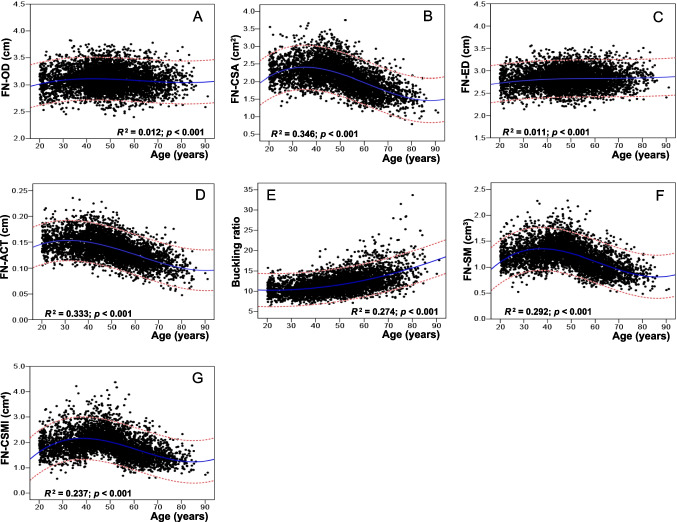
Scatter plots and best fitting curves and 95% confidence interval of age-related changes in FNGPs in Changsha-Chinese women. FN, femoral neck; OD, outer diameter; CSA, cross-sectional area; ACT, averaged cortical thickness; ED, endocortical diameter; SM, section modulus; CSMI, cross-sectional moment of inertia

Figure 1 shows the trends in the distributions, best fitting curve, and 95% CI of the FNGP plots of the Changsha-Chinese women by age. The best fit of the FNGPs variation with age was achieved by using the cubic regression model. However, the distribution of the scatter plots changed with the parameters, and the CSA, ACT, SM, and CSMI increased with age in the early stage and decreased with age after 40–49 years; only the trend in the distribution of the BR increased with age.

Table 3 shows the results of the comparisons of the overall mean FNGPs of the women of the different ethnic groups. The OD, ED, SM, and CSMI of the femoral neck in the Changsha-Chinese women were significantly lower than those of the NHWs by − 6.0% to − 24.4% (*p* = 0.025 to < 0.001). The CSA, ACT, SM, and CSMI of the Changsha-Chinese women were significantly lower than those of the NHBs by − 21.7% to − 25.0% (*p* = 0.030‒0.006), and their BR was significantly higher than that of the NHBs by 18.9% (*p* = 0.046). The relationship between the FNGPs and the ages of the women of the different ethnic groups in the US exhibited the best fit using the cubic regression model.

**Table 3:** Comparison of mean values of FNGPs in women of different races

FNGP	CCW (*n* = 4236)	NHWs (*n* = 3251)	NHBs (*n* = 2129)	MA (*n* = 1827)
Outer diameter (cm)	3.10 ± 0.07^a^	3.29 ± 0.09	3.17 ± 0.06	3.09 ± 0.05
Cross-sectional area (cm^2^)	2.01 ± 0.35^b^	2.27 ± 0.28	2.48 ± 0.35	2.21 ± 0.34
Endocortical diameter (cm)	2.84 ± 0.06^a^	3.01 ± 0.13	2.86 ± 0.08	2.81 ± 0.09
ACT (cm)	0.129 ± 0.022^b^	0.138 ± 0.023	0.157 ± 0.024	0.144 ± 0.024
Buckling ratio	12.7 ± 2.29^b^	12.2 ± 2.24	10.3 ± 1.68	11.1 ± 2.14
Section modulus (cm^3^)	1.12 ± 0.20^c^	1.33 ± 0.12	1.39 ± 0.17	1.21 ± 0.16
CSMI (cm^4^)	1.76 ± 0.33^c^	2.19 ± 0.17	2.20 ± 0.29	1.88 ± 0.25

Compared with NHWs, ^a^*p* < 0.001 and 0.003; compared with NHBs, ^b^*p* = 0.046‒0.013; compared with NHWs and NHBs, ^c^*p* = 0.025‒0.005

**Fig. 2:**
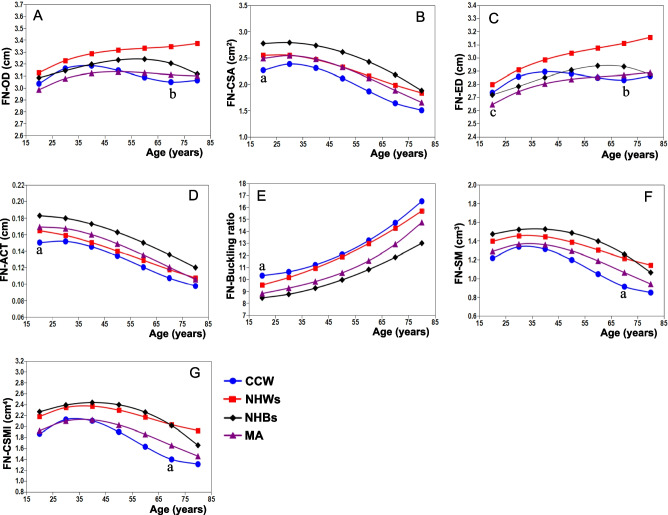
Comparison of fitting curves of age-related changes of FNGPs in Changsha-Chinese and American females. CCW, Changsha-Chinese women; NHWs, non-Hispanic Whites; NHBs, non-Hispanic Blacks; MA, Mexican Americans; FN, femoral neck; FNGPs, femoral neck geometric parameters; OD, outer diameter; CSA, cross-sectional area; ACT, averaged cortical thickness; ED, endocortical diameter; SM, section modulus; CSMI, cross-sectional moment of inertia. Compared with NHWs, NHBs, and MA in the same parameter, ^a^*p* = 0.002 to < 0.001; compared with NHWs, ^b^*p* < 0.001; compared with CCW, ^c^*p* = 0.042

Figure 2 shows that the reference curves of the CSA, ACT, SM, and CSMI of the Changsha-Chinese women were significantly lower than those of the NHWs, NHBs, and Mexican women (*p* = 0.002 to < 0.001). The OD and ED reference curves of NHWs were significantly highest (all *p* < 0.001). The CSA (Fig. 2B), ACT (Fig. 2D), and SM (Fig. 2F) reference curves of the Changsha-Chinese women were the lowest across the age range, while the BR reference curves were the highest (Fig. 2E). The OD (− 2.1% to − 9.7%), CSA (− 6.9% to − 21.9%), and ACT (− 3.5% to − 10.1%) curves of the Changsha-Chinese women were significantly lower than those of the NHWs (Table 4) (all *p* < 0.001). The curves of the CSA (− 17.1% to − 32.9%), ACT (− 18.5% to − 26.4%) and SM (− 13.7% to − 37.7%) were significantly lower in the Changsha-Chinese women than they were in the NHBs (all *p* < 0.001). However, the BR curves of the Changsha-Chinese women were significantly higher than those of the NHWs (1.6 to 7.6%), NHBs (17.3 to 21.3%) and Mexican women in the US (10.7 to 14.5%) (all *p* < 0.001).

**Table 4:** Differences in FNGPs between Changsha-Chinese women and American females with age

Age (yr)	OD differences (%)			CSA differences (%)			ED differences (%)			ACT differences (%)			BR differences (%)			SM differences (%)			CSMI differences (%)		
	A^a^	B^b^	C	A^a^	B^a^	C^a^	A^a^	B	C^b^	A^a^	B^a^	C^a^	A^a^	B^a^	C^a^	A^a^	B^a^	C^a^	A^a^	B^a^	C^b^
20–29	− 3.0	− 1.6	1.7	− 12.6	− 22.5	− 9.9	− 2.3	0.6	3.3	− 9.5	− 21.7	− 12.4	7.6	18.0	14.5	− 14.7	− 20.9	− 5.8	− 16.9	− 21.5	− 2.9
30–39	− 2.1	0.6	2.7	− 7.1	− 17.1	− 6.7	− 1.8	2.6	4.0	− 4.7	− 18.5	− 10.1	4.4	17.6	12.8	− 8.8	− 13.7	− 2.1	− 10.4	− 12.4	1.3
40–49	− 3.2	− 0.4	1.9	− 6.9	− 18.5	− 7.3	− 3.1	1.5	3.1	− 3.5	− 18.9	− 10.0	2.4	17.3	12.4	− 10.1	− 16.2	− 3.5	− 12.8	− 15.9	− 0.9
50–59	− 5.4	− 2.8	0.4	− 10.2	− 23.6	− 10.1	− 5.4	− 0.9	1.5	− 4.5	− 21.5	− 11.1	1.6	17.5	12.6	− 15.8	− 24.0	− 8.0	− 20.9	− 26.1	− 6.6
60–69	− 7.9	− 4.9	–1.3	− 15.6	− 30.0	− 13.5	− 7.9	− 3.2	− 0.3	− 7.0	− 24.7	− 12.3	1.9	18.2	12.7	− 24.6	− 33.4	− 13.5	− 33.2	− 38.6	− 13.9
70–79	− 9.7	− 5.1	–2.0	− 21.0	− 32.9	− 14.8	− 9.8	− 3.6	− 1.3	− 9.6	− 26.4	− 12.2	3.1	19.5	12.2	− 33.1	− 37.7	− 16.5	− 45.5	− 44.3	− 18.4
≥ 80	− 9.7	− 1.4	–0.9	− 21.9	− 24.8	− 9.8	− 10.3	− 0.5	− 0.9	− 10.1	− 22.9	− 8.3	4.9	21.3	10.7	− 34.5	− 25.2	− 10.7	− 47.0	− 26.6	− 11.2

A = Changsha-Chinese women (CCW) vs non-Hispanic Whites (NHWs); B = CCW vs non-Hispanic Blacks (NHBs); C = CCW vs Mexican Americans (MA); Differences (%) = (CCW − female of other race)/CCW × 100

The difference in the reference curve comparison between Changsha-Chinese women and female of other races in the same indicator, ^a^*p* < 0.001 and ^b^*p* = 0.042–0.002

## Discussion

This comparative study confirmed ethnic differences in FNGPs between Changsha-Chinese women and American NHWs, NHBs, and Mexican women. The CSA, SM, and CSMI of the femoral necks of the Changsha-Chinese women were smaller, the ACT was thinner, and the BR was higher, suggesting that the mechanical strength of the femoral necks of the Chinese women were weaker and the ability to resist bending and torsional stress was compromised. Based on these geometric parameters, one could predict that Chinese women have a higher risk of hip fracture than American women have. However, numerous studies have shown that Chinese and other Asian women have very low hip-fracture rates compared to those of White American women [24–29]. This paradoxical phenomenon suggests that many other factors other than bone structural geometry are associated with the risk of hip fracture. Early studies by Lauderdale et al. [27] showed that the hip fracture rate of Asian American (Chinese and Japanese) women was lower than that of White American women because the shorter hip axis and femoral neck and the larger femoral neck angle of Asian women [30, 31] reduced the risk of hip fracture. Compared with the hip axis and femoral neck of White British women, those of Shenyang-Chinese women were found to be shorter, and the femoral neck’s OD, CSA, CSMI, and SM were smaller [32]. Even after adjusting for height and weight, the differences between the two samples remained. Therefore, the shorter hip axis and femoral neck of Chinese women may be a protective factor that accounts for their lower hip-fracture rate. Compared with White Australian women, women in Beijing, China, have a lower OD and BR of the femoral neck and a higher SM [33], suggesting that such differences have a protective effect on the femoral neck of Beijing women in China by increasing its resistant to destruction, thereby lowering their hip-fracture rate. Another study showed that although the ODs and SMs of the femoral necks of Chinese women were lower than those of White American women [34], the compressive strength index (CSI) of Chinese women, which integrates information related to BMD, OD, and body weight, remained significantly higher than that of White American women. Therefore, a higher CSI may be an important reason for the lower hip-fracture rate of Chinese women [34]. A bone microstructure analysis showed that, compared with White women, Chinese-American women had an 80–95% higher plate volume fraction, an 18–20% higher plate number density and a twofold higher plate-to-rod ratio with more axial trabecular networks [28, 35]. These findings indicate that Chinese people with a thicker cortex and more plate-like bones had a lower risk of flexion, which may have helped to reduce their risk of hip fracture [28]. The lower rate of hip fracture in mainland China may also be related to their traditional lifestyle. The elderly tend to live with their adult children who accompany them on a daily basis, which may reduce the risk of fall-related fractures in the elderly [36]. Recent studies have found that a low body weight, less physical activity, diabetes, prior fractures, or self-rated poor health [37], as well as menopause at a younger age, longer postmenopausal intervals, or a lower total of reproductive years, are associated with an increased risk of hip fracture in Chinese women [38]. Older women living near major roads also have an increased risk of osteoporotic fractures associated with traffic pollution [39].

The present study confirmed ethnic differences in the reference curves of the FNGPs between the Changsha-Chinese women and ethnic groups of the US women. The reference curves of the CSA, ACT, and SM of the Changsha-Chinese women were lower than those of the NHWs, NHBs, and Mexican women across the entire range of ages, although the BR reference curves were higher than those of the three ethnic groups of US women. According to the reference curves, the CSA, ACT, SM, and CSMI of the Changsha-Chinese women were highest at age 30 and the OD and ED at the age of 40, after which they gradually declined with age. The changing trends of these curves differed from those of the three ethnic groups of US women. In particular, the OD (Fig. 2A) and ED (Fig. 2C) reference curves of the NHWs always showed an increased trend with an increase in age. The reference curves of all four ethnic groups of women in this study were very similar for ACT and BR. By age 80, the cumulative reduction of ACT in the Changsha-Chinese women was 35.7% (results not shown), and 34.7%, 34.4%, and 37.5%, respectively, in the NHWs, NHBs, and Mexican women. The cumulative BR increase in the Changsha-Chinese women was 60.1%, and it was 64.8%, 53.8%, and 67.1%, respectively (results not shown), in the NHWs, NHBs, and Mexican women. In a cross-sectional study by Kang et al. [40], it was found that the CSA, ACT, and SM of the femoral neck decreased by 0.75%, 1.24%, and 0.64%, respectively, and the OD and BR increased by 0.16% and 1.34%, respectively, with a 1-year increase in age among Chinese postmenopausal women. Mayhew et al. [41] observed that, compared with age 60, the thickness of the femoral neck cortex and the critical stress of the femur decreased by 6.4% per decade and 13.2% per decade, respectively. The cortical thickness of the femur became thinner, which weakened its ability to absorb energy, which, in turn, led to an increased risk of hip fracture. Another study showed that compared with the peripheral diameter of the femoral neck of Japanese women, those of American White women expanded faster and their buckling ratios increased significantly, as they aged, while the change rate of their CSAs and SMs was slower [42]. A recent study found that during the 10 years spanning the menopausal transition period, the CSA and SM decreased significantly and the OD increased significantly in the femoral necks of American women, with the largest reduction in CSA occurring among Japanese American women, rather than White, Black, or Chinese American women. Compared with White American women, the cumulative increase in the rate of the femoral neck’s OD in Black women was significantly lower [43]. This study also found that the OD of the femoral neck of the NHWs in the US increased with age more so than the OD of the NHBs (Fig. 2A), especially after age 60, when the OD of the NHWs continued to increase with age, whereas the OD of the NHBs decreased with age, indicating two completely opposite trends.

This study found ethnic differences in the FNGP reference curve of women, and other studies have found racial associations between FNGPs and hip-fracture risk in women [10, 12, 44–46]. Hip-fracture risk was found to be associated with an increased femoral neck OD among women in Spain [44] and Malaysia [45]. Our studies have shown that the CSA, ACT, SM, and CSMI of female patients with femoral neck fractures decreased, whereas the OD, ED, and BR increased. After adjusting for femoral neck BMD, age, height, weight, and BMI, the femoral neck ACT decrease and BR increase were independent risk factors for femoral neck fractures among women in Changsha, China [10]. Kaptoge et al. [46] conducted a 13-year follow-up study on American women, showing that the CSA, ACT, CSMI, and SM of the femoral neck of patients with hip fractures decreased, while their OD, ED, and BR increased. After adjusting for various potential factors, the decreases in ACT and SM were independently associated with the risk of hip fracture. Han et al. [12] reported that the CSA of the femoral neck decreased and the OD increased in female Korean patients with a femoral neck fracture. After adjusting for age, height, weight, and hip BMD, the risk of a femoral neck fracture increased by 1.97 times and 1.53 times with each standard deviation (SD) decrease in the CSA and each SD increase in the OD, respectively. These findings suggest that the bone structural parameters that predict the risk of hip fracture may vary by ethnicity or may be related to the characteristics of the study sample and the statistical analysis model that is used.

This study has limitations. First, as described by other researchers [20, 47], the three-dimensional model of the femoral neck cross-section area derived from the DXA 2D images might not fully conform to the bone geometry of individual subjects, thereby affecting the accuracy of the FNGP estimates. Second, the bone geometry measured by DXA can only evaluate the macro structure of the entire cross-sectional area, and it cannot evaluate the microscopic structure of thinning osteoporotic bones [48]. Third, simulating the cortical bone at the narrowest point of the femoral neck as a uniformly thick circular ring may not be consistent with the actual state of the bone. However, compared with the 3D measurement technology of high-resolution quantitative computed tomography, it is easier to obtain bone structure parameters by 2D or 3D analysis of DXA scan images, and this has been supported by the bone geometric parameters obtained by two measurement techniques that were highly correlated [49, 50].

## Conclusion

This study is the first to report ethnic differences in age-related reference curves of FNGPs between Changsha-Chinese women and non-Hispanic Whites, non-Hispanic Blacks, and Mexican women in the US. The differences varied by race, age, and measurement parameters, with the most significant features being the CSA, ACT, SM, and CSMI reference curves of the femoral necks of Changsha-Chinese women, which were significantly lower than those of non-Hispanic Whites, non-Hispanic Blacks, and Mexican women in the US, although the BR reference curves were completely reversed. Based on these differences in FNGPs, it should be possible to explain differences in hip-fracture rates between ethnic groups.

## Supplementary Information

Below is the link to the electronic supplementary material.

Supplementary file1 (DOCX 228 KB)

## Data Availability

The original contributions presented in the study are included in the article. Further inquiries can be directed to the corresponding author.
