# Supplementary material for: Comparisons of reference curves for femoral neck geometric parameters between Changsha-Chinese women and women of three ethnic groups in the United States
Source: Arch Osteoporos. 2025 Jul 8;20(1):86. doi: 10.1007/s11657-025-01571-y (PMC12238157; doi:10.1007/s11657-025-01571-y)
Supplement: Supplementary file 1 — Supplementary file1 (DOCX 228 KB) [file 11657_2025_1571_MOESM1_ESM.docx]

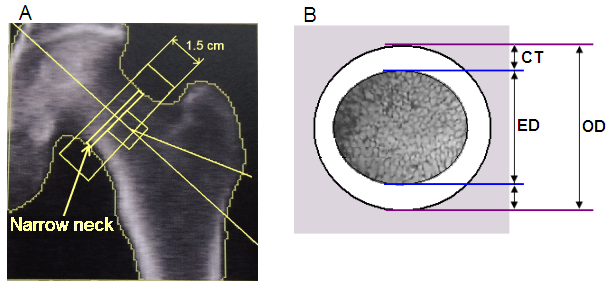

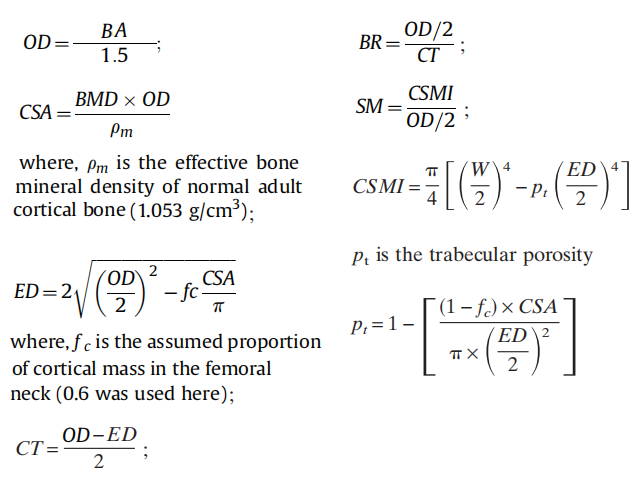


**Fig. S1** FNGPs anatomical location diagram and calculation equations. (A) Hologic scanner measures the proximal femur image and FNGP measurement position. (B) Schematic diagram of the cross-section at the narrow part of the femoral neck. OD, outer diameter, also known as width (W); BA, bone area; CSA, cross-sectional area; ED, endocortical diameter; ACT, averaged cortical thickness; BR, buckling ratio; SM, section modulus; CSMI, cross-sectional moment of inertia.
